# Frailty as a Key Determinant of Cardiovascular Risk and Mortality in Preserved Ratio Impaired Spirometry: A Nationally Representative Study

**DOI:** 10.1111/crj.70165

**Published:** 2026-01-10

**Authors:** Yue Ren, Yixing Wu, Weiping Hu, Li Liu, Hui Cai, Jing Zhang

**Affiliations:** ^1^ Department of Pulmonary and Critical Care Medicine, Zhongshan Hospital, Shanghai Medical College Fudan University Shanghai China

**Keywords:** cardiovascular disease, frailty, MACEs, mortality, PRISm

## Abstract

**Background:**

Preserved ratio impaired spirometry (PRISm) is associated with elevated cardiovascular disease (CVD) risk and progression to COPD, but the underlying mechanisms remain unclear. Frailty is known to worsen outcomes in COPD; however, its role in PRISm has not been well defined. This study examined factors associated with cardiovascular events and mortality in PRISm and developed risk models.

**Methods:**

We analyzed 8882 adults (aged 20–79 years) from NHANES 2007–2012, identifying 763 (8.6%) with PRISm (FEV_1_/FVC ≥ 0.70 and FEV_1_ < 80% predicted). Frailty was assessed using the 23‐item laboratory frailty index (FI‐LAB; cut‐off ≥ 0.23). The primary outcome was all‐cause mortality, obtained from linked National Death Index records; the secondary outcome was major adverse cardiovascular events (MACEs: myocardial infarction, stroke, heart failure, or angina), assessed cross‐sectionally. LASSO regression and multivariable logistic/Cox models were used to identify variables independently associated with the outcomes, and nomograms were constructed.

**Results:**

PRISm participants had higher frailty prevalence (53.9% vs. 45.5%) and more MACEs (16.2% vs. 6.0%) than those with normal spirometry (both *p* < 0.0001). Frailty was independently associated with prevalent MACEs (adjusted OR = 18.87, *p* < 0.001) and was bidirectionally associated with PRISm (OR = 1.40, *p* < 0.001). Key factors independently associated with MACEs included frailty index, age, sex, anemia, and emphysema (AUC = 0.786). Over 9.9 years, mortality was higher in frail vs. non‐frail PRISm individuals (15.2% vs. 7.0%; adjusted HR = 30.66). Frailty severity demonstrated a clear mortality gradient, and a mortality nomogram integrating age and frailty achieved an AUC of 0.81.

**Conclusion:**

Frailty is strongly and independently associated with cardiovascular morbidity and mortality. FI‐LAB offers a practical tool for risk stratification and may help guide targeted preventive strategies.

## Graphical abstract

In adults with preserved ratio impaired spirometry (PRISm), frailty was common and strongly associated with increased cardiovascular events and all‐cause mortality. Frailty significantly improved risk stratification beyond traditional clinical factors. A frailty‐based nomogram demonstrated good discrimination and calibration, highlighting frailty as a key determinant of adverse outcomes in PRISm.

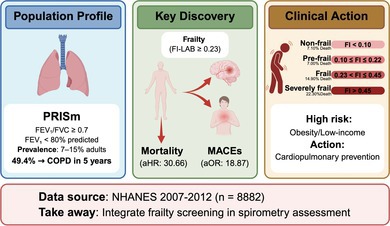

**Abbreviations**
ATS: The American Thoracic Society
AUC: time‐dependent receiver operating characteristic curve
BMI: body mass index
CI: confidence interval
CVD: cardiovascular disease
DCA: decision curve analysis
FEV_1_
: forced expiratory volume in 1 s
FI: frailty index
FI‐LAB: frailty index composed of routine laboratory data
FVC: forced vital capacity
HF: heart failure
HR: hazard ratio
LDH: lactate dehydrogenase
MACEs: major adverse cardiovascular events
MI: myocardial infarction
NCHS: National Center for Health Statistics
NHANES: National Health and Nutrition Examination Survey
OR: odds ratio
PEF: peak expiratory flow
PHQ‐9: The Patient Health Questionnaire 9
PIR: poverty–income ratio
PRISm: preserved ratio impaired spirometry
ROC: receiver operating characteristic

## 1 Introduction

Preserved ratio impaired spirometry (PRISm), characterized by reduced forced expiratory volume in 1 s (FEV_1_) and forced vital capacity (FVC) with a preserved FEV_1_/FVC ratio ≥ 0.7, represents a clinically significant yet underrecognized pulmonary phenotype [1, 2, 3, 4]. Global estimates indicate that PRISm affects 7%–15% of adults, with disproportionately higher prevalence in low‐ and middle‐income countries (LMICs) [5, 6]. Notably, a longitudinal study revealed that up to 49.4% of PRISm individuals progress to chronic obstructive pulmonary disease (COPD) within 5 years, while 15.7% revert to normal spirometry, underscoring its role as a critical precursor to COPD [7]. PRISm contributes substantially to the growing global burden of COPD‐related morbidity and mortality, now the third leading cause of death worldwide [8, 9]. Parallel to COPD [10, 11], PRISm has been identified as an independent risk factor for cardiovascular disease (CVD) incidence and mortality [7, 12]. However, the specific factors driving CVD risk in PRISm individuals remain poorly understood, limiting the development of targeted prevention strategies.

Frailty—a syndrome of multisystem physiological decline characterized by reduced resilience to stressors—is highly prevalent in older adults and strongly predicts adverse outcomes including hospitalization and mortality [13]. Its prevalence is markedly higher in COPD than in healthy age‐matched populations, where it is associated with increased exacerbations, reduced physical function, and impaired quality of life [10, 14, 15]. Frailty and COPD are thought to share biological pathways such as chronic systemic inflammation, oxidative stress, and mitochondrial dysfunction, all of which contribute to skeletal muscle impairment and cardiopulmonary vulnerability [16, 17]. Despite these insights, the relationship between frailty and PRISm—an at‐risk population that bridges respiratory impairment and cardiometabolic morbidity—has not been previously examined.

Addressing this gap is critical because frailty may represent a modifiable and clinically actionable determinant of outcomes in PRISm. Using nationally representative data from the US National Health and Nutrition Examination Survey (NHANES), this study aimed to examine factors independently associated with cardiovascular events and all‐cause mortality in individuals with PRISm and to assess their prognostic value through the development of individualized risk models.

## 2 Methods

### 2.1 Study Population

Data were obtained from the 2007–2012 National Health and Nutrition Examination Survey (NHANES), which employs a complex, stratified, multistage probability sampling design to generate a nationally representative sample of non‐institutionalized US adults. All NHANES participants provided written informed consent, and all data are publicly available without personal identifiers; therefore, this study was exempt from institutional review board approval.

Individuals aged 20–79 years with valid spirometry and complete information required for lung function predictive equations (age, sex, height, race) were eligible. Spirometry quality for FEV_1_ and FVC was graded from A to F according to American Thoracic Society (ATS) criteria, and only tests graded A or B were included. Participants were excluded if they had (1) FEV_1_/FVC < 0.70; (2) missing mortality data; (3) insufficient data to calculate FI‐LAB (< 70% of lab variables available); (4) pregnancy; or (5) self‐reported AIDS. A total of 8882 participants met the inclusion criteria (Figure S1).

### 2.2 Lung Function Measurement and PRISm Definition

Spirometry was performed at the NHANES Mobile Examination Centers following standardized ATS protocols [18]. To inclusively expand our analytical cohort, the diagnosis PRISm was ascertained utilizing pre‐bronchodilator spirometry. This approach aligns with methodologies employed in the existing literature, ensuring a comparable and scientifically rigorous analysis [19].

The predicted values of FEV_1_ and FVC were calculated using the Global Lung Function Initiative's (GLI) race‐neutral predictive equation [20]. PRISm was defined as recommended by the Global Initiative for Chronic Obstructive Lung Disease (GOLD) as FEV_1_/FVC ≥ 0.7 and FEV_1_ < 80% [21]. Normal spirometry (NS) was defined as FEV_1_ of ≥ 80% predicted with FEV_1_ to FVC ratio of ≥ 0.70.

### 2.3 Clinical and Laboratory Evaluations

NHANES collected detailed demographic, socioeconomic, behavioral, medical history, physical examination, and laboratory data. Covariates included age, sex, race/ethnicity, education, smoking status, serum cotinine, alcohol consumption, physical activity, body mass index (BMI), household income, and comorbidities.

Poverty–income ratio (PIR) was used to estimate socioeconomic status. We categorized PIR as low (PIR < 1, below federal poverty threshold), middle (PIR 1–< 4), and high (PIR ≥ 4) [22]. The age groups were 20–39, 40–59, and ≥ 60 years. BMI categories followed WHO adult BMI classification: underweight (BMI < 18.5), normal weight (18.5 ≤ BMI < 25), overweight (25 ≤ BMI < 30), and obese (BMI ≥ 30) [23]. Smoking status was categorized as current smokers (smoked 100 cigarettes and currently smoke), former smokers (smoked 100 cigarettes and quit), and never‐smokers (smoked less than 100 cigarettes). Alcohol consumption was categorized into never‐drinkers, non‐drinkers (no alcohol in the last year), moderate drinkers (up to 1 drink/day for women, up to 2 drinks/day for men), and heavy drinkers (more than 1 drink/day for women, more than 2 drinks/day for men) [24].

### 2.4 Definition of Frailty

Frailty was determined using the FI‐LAB [25], which comprises 23 physiological variables, including 21 routine blood tests plus systolic and diastolic blood pressure. Each variable was coded as 1 (abnormal) or 0 (normal). FI‐LAB scores were calculated only for participants with ≥ 70% of laboratory items available. The index is the ratio of abnormal variables to the total considered, ranging from 0 to 1, with higher scores indicating greater frailty [26]. A threshold of FI‐LAB ≥ 0.23 was used to define frailty [27].

### 2.5 Outcomes

The primary outcome was all‐cause mortality, ascertained through linkage with the National Death Index through December 31, 2019. Follow‐up time was calculated in months from the NHANES examination date to death or censoring. Secondary outcomes included major adverse cardiovascular events (MACEs)—a composite of nonfatal myocardial infarction, nonfatal ischemic stroke, heart failure, and angina—derived from standardized NHANES medical history questionnaires. Because NHANES does not longitudinally adjudicate cardiovascular events, MACE components were assessed cross‐sectionally, whereas mortality analyses utilized prospective follow‐up data.

### 2.6 Statistical Analysis

Given the complex multistage sampling design of NHANES, all analyses incorporated appropriate examination weights to ensure national representativeness of the US civilian noninstitutionalized population. The WTMEC2YR variable was applied for weighted analyses, and sampling weights were recalibrated according to NHANES analytic guidelines to account for the combined three survey cycles [28]. Continuous variables were presented as mean ± standard deviation (SD) for normally distributed data or median with interquartile range (IQR) for skewed distributions, while categorical variables were summarized as weighted proportions with 95% confidence intervals (CIs). Group differences were assessed using the chi‐square test for categorical variables; the independent Student's *t*‐test or one‐way ANOVA for normally distributed continuous variables; and the Mann–Whitney *U* test or Kruskal–Wallis test for non‐normally distributed variables.

To identify key variables associated with the outcome, LASSO regression was applied, which is well‐suited for high‐dimensional data and variable selection. The optimal penalty parameter (λ) was determined via 10‐fold cross‐validation. Laboratory biomarkers identified by LASSO, together with clinical and demographic variables that were significant in univariate analyses, were subsequently included in multivariable logistic or Cox proportional hazards regression models according to the type of outcome (cross‐sectional MACEs vs. longitudinal mortality). Only variables that remained significant at *p* < 0.05 in both univariate and multivariable models were considered independent risk factors and incorporated into the final nomograms. Model discrimination was evaluated using receiver operating characteristic (ROC) curve analysis, while internal validation and robustness were examined using 1000 bootstrap resamples. Clinical utility was assessed with decision curve analysis (DCA), which quantified the net benefit across a range of threshold probabilities. All statistical tests were two‐tailed, and a significance level of *p* < 0.05 was applied. Analyses were conducted using R software (version 4.2.0; R Foundation for Statistical Computing, Vienna, Austria) and SPSS Statistics version 22 (IBM Corp., Armonk, NY, USA).

### 2.7 Sensitivity Analyses

Several sensitivity analyses were performed to evaluate the robustness of our findings. First, alternative frailty index (FI) thresholds were applied to account for heterogeneity in frailty definitions. Cut‐off Set 1 categorized individuals as frail (FI ≥ 0.25), mildly frail (0.08 < FI < 0.25), or non‐frail (FI ≤ 0.08), while Cut‐off Set 2 classified participants as severely frail (FI > 0.45), frail (0.23 < FI ≤ 0.45), pre‐frail (0.10 ≤ FI ≤ 0.22), or non‐frail (FI < 0.10). Second, subgroup analyses were conducted across strata of gender, smoking status, age, BMI, and PIR to assess whether associations between frailty, PRISm, and adverse outcomes were consistent across demographic and clinical subgroups. Finally, to address potential misclassification related to the fixed‐ratio spirometric definition of PRISm, all primary analyses were repeated using lower limit of normal (LLN) criteria derived from NHANES III reference equations. Individuals meeting LLN‐defined PRISm criteria were re‐evaluated for associations with MACEs and all‐cause mortality using identical modeling strategies. These LLN‐based analyses assessed the stability of frailty‐associated risk estimates under an alternative, physiologically grounded spirometric definition.

## 3 Results

### 3.1 Baseline Characteristics by Spirometric Pattern

A total of 8882 adults were included after applying eligibility criteria (Figure S1). Among them, 763 participants (8.6%) had PRISm, while 8119 (91.4%) exhibited normal spirometry (NS) (Table 1).

**TABLE 1:** Baseline characteristics of the study population by spirometric pattern.

Characteristics	Total (*N* = 8882)	NS (*N* = 8119)	PRISm (*N* = 763)	*p*
Weighted population	124 585 152	117 554 976	7 030 176	—
Age (years)	47.2 ± 14.6	42.9 ± 14.5	47.8 ± 14.5	< 0.0001
Female (%)	52.2	52.0	56.3	< 0.0001
BMI (kg/m^2^)	28.8 ± 6.6	28.7 ± 6.4	31.9 ± 8.7	< 0.0001
Race, %				< 0.0001
Mexican American	9.2	9.6	2.7	
Other Hispanic	5.9	6.1	3.0	
Non‐Hispanic White	68.0	69.8	38.7	
Non‐Hispanic Black	10.2	8.1	45.1	
Other race	6.7	6.4	10.5	
Education, %				< 0.0001
Less than high school	15.7	15.5	19.8	
High school or equivalent	21.0	20.7	26.3	
Greater than high school	63.3	63.9	53.9	
Marital status, %				< 0.0001
Married	55.9	56.4	48.3	
Widowed/divorced/separated	15.2	14.7	24.6	
Never married	20.8	20.8	20.6	
Living with partner	8.0	8.1	6.5	
Poverty–income ratio (PIR)	3.1 ± 1.7	3.1 ± 1.7	2.6 ± 1.7	< 0.0001
Lung function				
FVC (mL)	4168.9 ± 1065.3	4241.6 ± 1033.8	2952.8 ± 839.3	< 0.0001
FEV_1_ (mL)	3338.2 ± 867.2	3400.8 ± 839.3	2292.3 ± 623.3	< 0.0001
Frailty index	0.22 ± 0.09	0.22 ± 0.08	0.24 ± 0.09	< 0.0001
Frailty prevalence (%)	46.0	45.5	53.9	< 0.0001
Smoking status, %				< 0.0001
Never smoker	58.6	59.0	51.7	
Former smoker	19.3	19.0	24.5	
Current smoker	22.1	22.0	23.8	
General health condition, %^a^				< 0.0001
Excellent	18.9	19.4	11.1	
Very good/good	67.6	68.0	62.4	
Fair	11.7	11.1	21.1	
Poor	1.8	1.5	5.4	
Mortality (%)	4.2	3.7	11.4	< 0.0001
MACEs (%)	6.6	6.0	16.2	< 0.0001

*Note:* Weighted population estimates reflect NHANES sampling weights (WTMEC2YR) recalibrated across 2007–2012 cycles.

Abbreviations: BMI, body mass index; FEV_1_, forced expiratory volume in 1 s; FVC, forced vital capacity; MACEs, major adverse cardiovascular events; NS, normal spirometry; PIR, poverty–income ratio; PRISm, Preserved Ratio Impaired Spirometry.

^a^ General health condition was self‐reported using standardized NHANES questionnaires.

Compared with NS individuals, those with PRISm were older, more often female, and more frequently non‐Hispanic Black. They had lower education levels, were less likely to be married, and demonstrated greater socioeconomic disadvantage. PRISm participants also had a higher prevalence of smoking, elevated serum cotinine levels, and substantially worse lung function, including lower FVC, FEV_1_, and PEF. Physical activity levels and alcohol consumption were lower in the PRISm group. Although many laboratory indicators differed statistically between groups, most showed limited clinical relevance.

PRISm participants had higher rates of obesity, poorer self‐reported health, increased healthcare utilization, and more comorbidities—including hypertension, diabetes, anemia, gout, arthritis, emphysema, thyroid and liver disorders, chronic bronchitis, depression, and cancer. Cardiovascular conditions were notably more prevalent in PRISm than NS, including MACEs (16.2% vs. 6.0%), heart failure (4.5% vs. 0.8%), angina (3.3% vs. 1.2%), myocardial infarction (4.4% vs. 1.4%), and stroke (2.8% vs. 1.2%) (all *p* < 0.0001) (Tables 1 and S1).

### 3.2 Frailty Burden and Clinical Correlates in PRISm

Frailty was more common and more severe in individuals with PRISm. FI‐LAB scores were higher in PRISm than NS (0.24 ± 0.09 vs. 0.22 ± 0.08; *p* < 0.0001), and frailty prevalence was greater (53.9% vs. 45.5%; *p* < 0.0001) (Table 1). Within the PRISm group, frail individuals were older (49.4 vs. 45.9 years), predominantly female (63.8%), and disproportionately non‐Hispanic Black (49.2%). Frailty was associated with worse cardiopulmonary profiles, including higher BMI (+1.7 kg/m^2^), smoking history, and reduced FEV_1_ (−10.8%), FVC (−10.8%), and PEF25–75 (−11.3%) (all *p* < 0.0001).

Frail PRISm individuals had substantially higher rates of cardiovascular diseases than non‐frail counterparts: MACEs (21.6% vs. 9.7%), myocardial infarction (6.1% vs. 2.3%), heart failure (6.4% vs. 2.4%), coronary heart disease (5.8% vs. 2.9%), angina (5.2% vs. 1.0%), and stroke (4.1% vs. 1.3%) (all *p* < 0.0001). Frailty was also associated with a higher prevalence of hypertension, diabetes, obesity, and increased healthcare utilization (Table S2).

### 3.3 Bidirectional Association Between PRISm and Frailty

A bidirectional relationship was observed between PRISm and frailty. PRISm prevalence was higher among frail individuals than non‐frail individuals (6.6% vs. 4.8%; *p* < 0.0001). In multivariable analyses, PRISm remained independently associated with frailty (Model I: OR 1.11; Model II: OR 1.06; both *p* < 0.001). Conversely, frailty was also independently associated with PRISm (Model I:OR 1.13; Model II:OR 1.05;both *p* < 0.001), and this association persisted after full adjustment (Table S3).

### 3.4 Cardiovascular Outcomes and Risk Modeling in PRISm

LASSO regression identified candidate variables associated with MACEs from 43 variables (Figure S2). These variables, along with clinically relevant covariates from univariate analyses, were included in multivariable logistic regression. Age (OR 1.071, *p* < 0.05), FI (OR 18.87, *p* < 0.001), gender (OR 1.791, *p* < 0.05), anemia (OR 3.004, *p* < 0.001), and emphysema (OR 8.937, *p* < 0.001) were independent risk factors (Table S4).

Three logistic regression models were constructed:

Model 1: LDH + FI (AUC 0.640)

Model 2: Gender + age + LDH + FI (AUC 0.779)

Model 3: Model 2 + anemia + emphysema (AUC 0.786)

**FIGURE 1:**
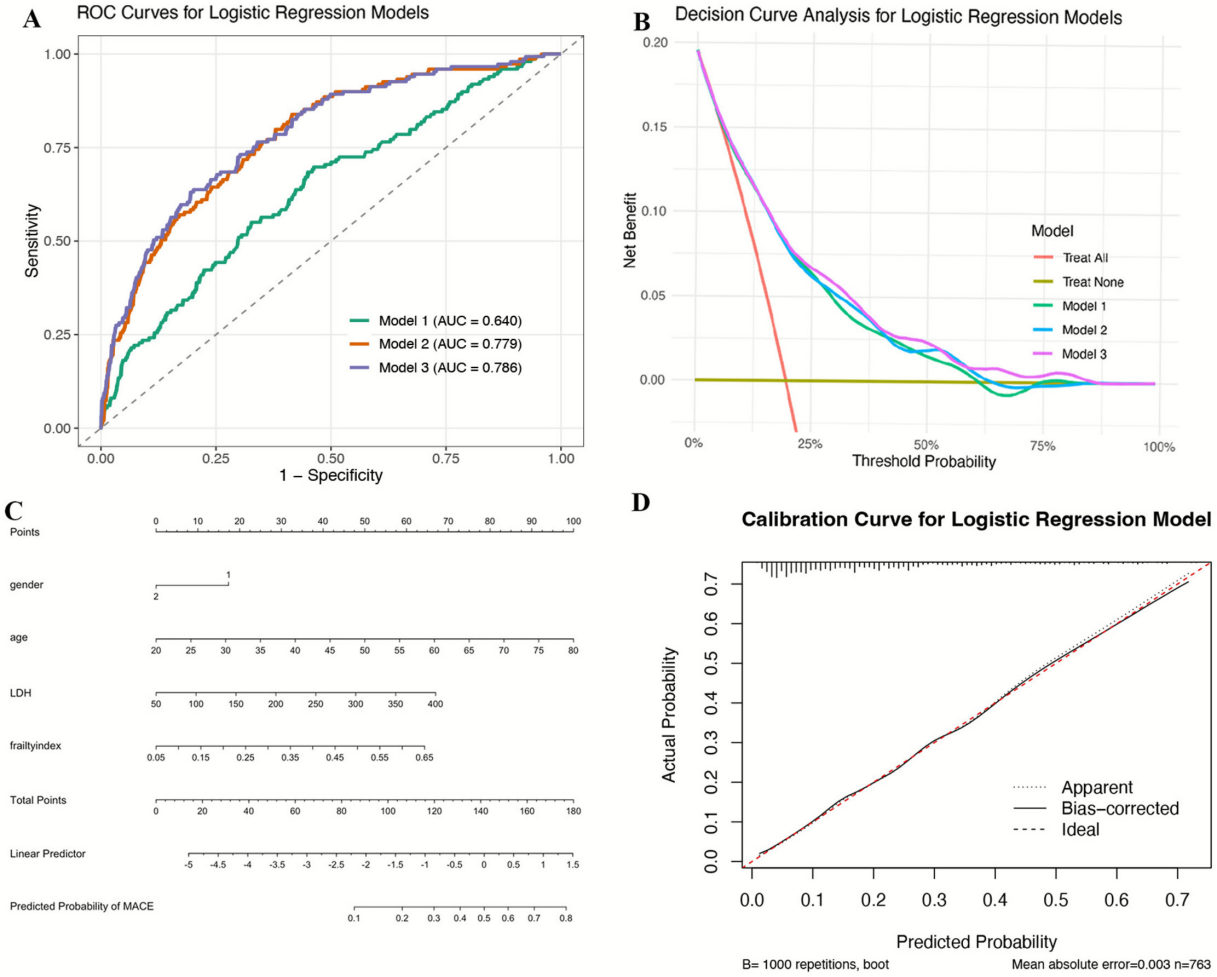
Discrimination and clinical utility of MACE risk models in PRISm. (A) Receiver operating characteristic (ROC) curves illustrating the discriminative performance of three logistic regression models for estimating the risk of major adverse cardiovascular events (MACE) in individuals with PRISm. Model 1 included lactate dehydrogenase (LDH) and frailty index (FI); Model 2 additionally incorporated age and sex; Model 3 further included anemia and emphysema. (B) Decision curve analysis demonstrating the net clinical benefit of each model across a range of threshold probabilities, compared with treat‐all and treat‐none strategies. (C) Nomogram derived from the multivariable logistic regression model for individualized risk estimation of MACE in PRISm. (D) Calibration curve comparing observed and model‐estimated MACE risk, with bias correction based on 1000 bootstrap resamples, showing good agreement.

Although Model 3 showed the highest AUC, Model 2 demonstrated the most favorable balance between discrimination and clinical utility on decision curve analysis and was therefore selected for the final nomogram (Figure 1A–C). Calibration curves demonstrated close agreement between predicted and observed risks (Figure 1D). Frailty remained significantly associated with MACEs, MI, heart failure, stroke, and angina across all adjustment models (OR range 2.56–5.44; all *p* < 0.001) (Table 2).

**TABLE 2:** Survey‐weighted logistic regression for the association between frailty and cardiovascular outcomes in PRISm.

Outcome	Frailty status	Unadjusted OR (95% CI)	Model 1 OR (95% CI)	Model 2 OR (95% CI)
MACEs	Non‐frail	Reference	Reference	Reference
	Frail	2.561 (2.549–2.572)	2.352 (2.341–2.364)	2.609 (2.594–2.624)
Myocardial infarction	Non‐frail	Reference	Reference	Reference
	Frail	2.703 (2.680–2.725)	2.159 (2.140–2.178)	1.643 (1.626–1.661)
Heart failure	Non‐frail	Reference	Reference	Reference
	Frail	2.781 (2.758–2.804)	2.275 (2.255–2.295)	2.248 (2.226–2.270)
Stroke	Non‐frail	Reference	Reference	Reference
	Frail	3.352 (3.315–3.389)	2.348 (2.322–2.375)	2.409 (2.379–2.440)
Angina	Non‐frail	Reference	Reference	Reference
	Frail	5.438 (5.374–5.502)	4.569 (4.513–4.625)	3.905 (3.851–3.960)

*Note:* Data are survey‐weight–adjusted odds ratios (ORs) with 95% confidence intervals (CIs). MACEs = major adverse cardiovascular events; Model 1: adjusted for sex, age, race/ethnicity, and body mass index (BMI). Model 2: additionally adjusted for education level, marital status, poverty–income ratio, smoking status, alcohol consumption, and physical activity.

### 3.5 Mortality and Survival Outcomes in PRISm

**FIGURE 2:**
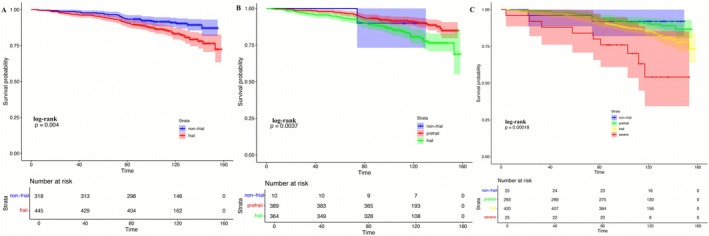
Kaplan–Meier survival curves according to frailty status in PRISm. (A) Overall survival comparing non‐frail and frail individuals with PRISm. (B) Overall survival across non‐frail, pre‐frail, and frail PRISm groups. (C) Overall survival stratified by four frailty categories (non‐frail, pre‐frail, frail, and severe frailty). Shaded areas represent 95% confidence intervals. *p* values were calculated using the log‐rank test. Numbers at risk are shown below each panel.

During a median follow‐up of 9.9 years, all‐cause mortality was substantially higher in individuals with PRISm compared with those with normal spirometry (11.4% vs. 3.7%) (Table 1). Within the PRISm cohort, frail participants experienced markedly greater mortality than non‐frail individuals (15.2% vs. 7.0%; *p* < 0.0001) (Table S2). Kaplan–Meier curves further demonstrated a clear, stepwise decline in survival with increasing frailty severity (Figure 2), with severely frail participants exhibiting the shortest median survival (118.2 months) compared with frail (143.2 months), pre‐frail (146.4 months), and non‐frail individuals (140.9 months).

LASSO Cox regression highlighted age and the frailty index as the principal mortality‐associated variables (Figure S3). In the multivariable Cox model, both older age (HR 1.05 per year, 95% CI 1.04–1.07) and higher frailty index (HR 30.66, 95% CI 4.14–225.82) remained independently associated with all‐cause mortality, together with a history of myocardial infarction (HR 2.08, 95% CI 1.18–3.69) and congestive heart failure (HR 2.81, 95% CI 1.64–4.83) (all *p* < 0.01; Table 3).

**TABLE 3:** Cox regression analysis of factors associated with all‐cause mortality in PRISm.

Variable	Univariate Cox regression			Multivariate Cox regression		
	HR	95% CI	*p*	HR	95% CI	*p*
Gender (female)	0.91	0.62–1.34	0.63	—	—	—
Age (per year)	1.065	1.048–1.082	< 0.001	1.053	1.037–1.070	< 0.001
BMI	0.996	0.972–1.020	0.72	—	—	—
Education			0.007	—	—	—
Less than high school	Reference	—			—	—
High school	0.544	0.320–0.926	0.02	—	—	—
Greater than high school	0.522	0.338–0.806	0.003	—	—	—
Frailty index	111.67	18.37–678.88	< 0.001	30.655	4.161–225.822	0.001
Hypertension	2.998	1.960–4.586	< 0.001	—	—	—
Hypercholesterolemia	1.697	1.132–2.543	0.01	—	—	—
Diabetes			< 0.001	—	—	—
Yes	2.594	1.745–3.857	< 0.001	—	—	—
Borderline	1.835	0.665–5.066	0.24	—	—	—
Arthritis	1.942	1.316–2.864	< 0.001	—	—	—
Gout	2.038	1.061–3.916	0.03	—	—	—
Congestive heart failure	6.145	3.833–9.849	< 0.001	2.814	1.641–4.825	< 0.001
Myocardial infarction	5.174	3.106–8.618	< 0.001	2.082	1.176–3.687	0.01
Stroke	3.749	1.949–7.213	< 0.001	—	—	—
Coronary heart disease	2.521	1.312–4.846	0.006	—	—	—
Angina	3.751	1.951–7.211	< 0.001	—	—	—
General health condition			< 0.001	—	—	—
Excellent	Reference	—	—	—	—	—
Very good/good	1.141	0.487–2.672	0.76	—	—	—
Fair	2.219	0.933–5.277	0.07	—	—	—
Poor	3.958	1.548–10.122	0.004	—	—	—

*Note:* Values are presented as hazard ratios (HRs) with 95% confidence intervals (CIs). Cox proportional hazards regression analyses were used to assess factors associated with all‐cause mortality in PRISm.

**FIGURE 3:**
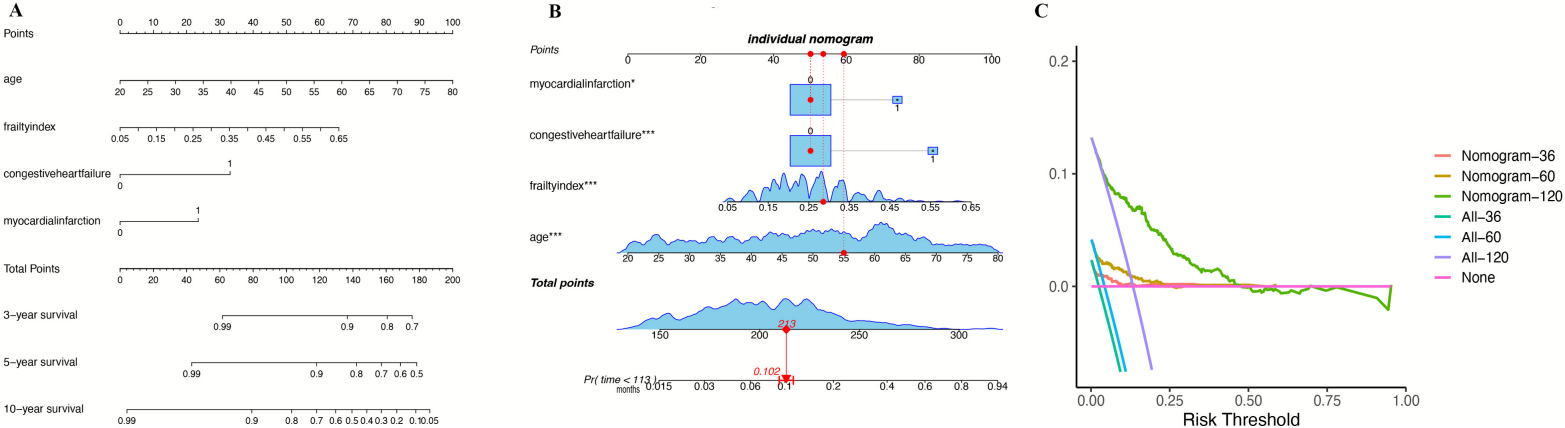
Nomogram development and clinical utility for mortality risk estimation in PRISm. (A) Nomogram incorporating age, frailty index, congestive heart failure, and myocardial infarction to estimate 3‐, 5‐, and 10‐year mortality risk in individuals with PRISm. (B) Example of individualized risk estimation using the nomogram, illustrating the calculation of total points and corresponding predicted mortality probability. (C) Decision curve analysis (DCA) evaluating the clinical net benefit of the nomogram at different time horizons (36, 60, and 120 months), compared with treat‐all and treat‐none strategies.

**FIGURE 4:**
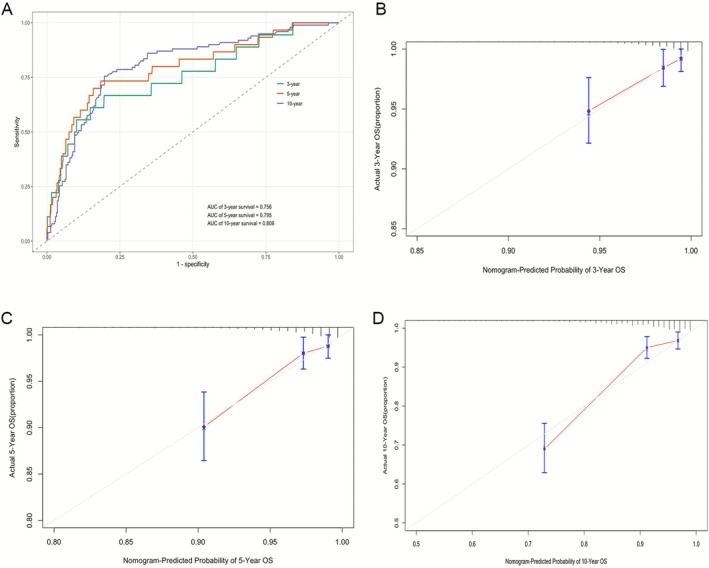
Discrimination and calibration of the mortality risk nomogram. (A) Time‐dependent ROC curves showing the discriminative performance of the nomogram for 3‐, 5‐, and 10‐year overall survival (OS). (B–D) Calibration plots comparing nomogram‐estimated and observed OS probabilities at 3 years (B), 5 years (C), and 10 years (D). The dashed line indicates ideal calibration, and points represent mean predicted probabilities with 95% confidence intervals.

These four variables were incorporated into a mortality risk nomogram (Figure 3A). As an illustrative example, a 55‐year‐old individual with PRISm, a frailty index of 0.29, and no history of cardiovascular comorbidities had an estimated 10.6% risk of death over 113 months, corresponding to the maximum duration of follow‐up in this cohort (Figure 3B). The model demonstrated good discriminative performance, with time‐dependent AUCs of 0.76, 0.80, and 0.81 for 3‐, 5‐, and 10‐year survival, respectively, and showed good calibration, with close agreement between predicted and observed mortality across risk strata (Figure 4).

### 3.6 Sensitivity and Subgroup Analyses

Subgroup analyses consistently identified high‐risk PRISm subpopulations. PRISm prevalence was highest among adults aged 40–59 years (44.6%), middle‐income individuals (53.2%), and those with obesity (55.4%). Frailty prevalence followed similar gradients. Mortality increased sharply with frailty severity (7.0% in pre‐frail vs. 22.3% in severely frail individuals), and associations held across gender, smoking status, and age strata (Figure S4; Tables S1, S5, and S6).

To address potential misclassification associated with fixed‐ratio spirometry, all primary analyses were repeated using LLN‐defined PRISm based on NHANES III equations. Associations between frailty and both MACEs and mortality remained robust in magnitude and direction, confirming that the prognostic importance of frailty is not dependent on the spirometric definition used (Tables S7–S9).

## 4 Discussion

This nationally representative analysis provides the first comprehensive evaluation of the interplay among frailty, PRISm, and cardiovascular outcomes in US adults. We demonstrate a strong bidirectional association between PRISm and frailty and identify frailty as a major independent determinant of both MACEs and all‐cause mortality in this population. These findings highlight frailty as a central, yet previously overlooked, component of risk stratification in individuals with impaired lung function who do not meet conventional COPD criteria.

Several patterns in our results underscore the clinical importance of frailty in PRISm. First, frailty prevalence exceeded 50% among individuals with PRISm—substantially higher than in those with normal spirometry. Second, frailty severity displayed a graded relationship with mortality, suggesting dose–response effects that reinforce its prognostic relevance. Third, frailty remained a dominant determinant associated with cardiovascular morbidity and long‐term mortality even after adjusting for demographic factors, comorbidities, lifestyle exposures, and lung function indices. Together, these observations indicate that frailty captures systemic vulnerability not reflected by spirometry alone and may help explain the disproportionately high cardiometabolic burden previously observed in PRISm cohorts.

The biological plausibility of these associations is supported by several converging mechanisms. Frailty is characterized by multisystem dysregulation—including chronic low‐grade inflammation, sarcopenia, mitochondrial dysfunction, impaired autonomic regulation, and reduced metabolic reserve—all of which may contribute to reduced lung compliance, diminished ventilatory efficiency, and accelerated pulmonary decline. PRISm itself is frequently associated with obesity, systemic inflammation, and metabolic disturbances, which overlap substantially with frailty phenotypes. The co‐occurrence of inflammation‐ and metabolism‐driven impairment likely amplifies cardiovascular vulnerability through endothelial dysfunction, atherogenesis, and heightened susceptibility to physiologic stress. In our cohort, elevated LDH levels in frail PRISm individuals further support the presence of cellular stress and metabolic strain [29]. These shared pathways offer a compelling mechanistic framework for the markedly elevated rates of MACEs and mortality observed in frail individuals with PRISm.

Beyond mechanistic insight, our risk modeling offers clinically actionable tools for risk stratification. Across all modeling approaches, the frailty index showed the strongest and most consistent association with cardiovascular morbidity and mortality, exceeding that of traditional cardiopulmonary variables. The resulting nomograms demonstrated robust discrimination and calibration, supporting their utility for individualized prognostication in routine clinical settings. Importantly, nearly half of PRISm individuals in our study were middle‐aged (40–59 years), underscoring the potential impact of early frailty detection and intervention during a window when preventive strategies may be most effective.

Our methodological approach also advances the operationalization of frailty for large‐scale health assessments. The FI‐LAB, derived exclusively from routine laboratory measures, provides a scalable and cost‐efficient alternative to phenotype‐based assessments. This is particularly relevant in low‐ and middle‐income countries—regions with disproportionately high PRISm prevalence—where resource constraints limit the feasibility of standardized physical performance testing [5]. Consistent results across multiple sensitivity analyses—including alternative frailty thresholds and LLN‐based PRISm definitions—reinforce the robustness and generalizability of our findings.

This study has limitations. Spirometry in NHANES was performed without bronchodilation, which may limit comparability with GOLD‐defined post‐bronchodilator PRISm/COPD classifications [30, 31]. Frailty measurements were available only at baseline, preventing assessment of temporal changes or causal directions in the frailty–PRISm relationship. While FI‐LAB is validated and correlates well with clinical frailty phenotypes [5, 16, 32, 33, 34, 35], integration of physical performance measures could further enhance characterization. Finally, MACE outcomes were assessed cross‐sectionally due to NHANES design constraints, limiting temporal inference for cardiovascular events. These limitations highlight the need for longitudinal cohorts incorporating repeated frailty assessments and post‐bronchodilator lung function to elucidate causal pathways and guide intervention development.

## 5 Conclusion

Frailty is a critical and independent determinant of cardiovascular morbidity and mortality in individuals with PRISm. The strong bidirectional relationship between frailty and PRISm underscores shared pathophysiological mechanisms and highlights frailty as a pivotal target for early risk assessment. The FI‐LAB–based nomograms developed in this study offer practical, scalable tools for personalized risk estimation, particularly valuable in resource‐limited settings where PRISm burden is greatest. These findings call for urgent clinical trials evaluating multimodal frailty interventions as a strategy to attenuate cardiopulmonary decline and reduce the global burden associated with this underrecognized respiratory phenotype.

## Author Contributions

J.Z. and Y.R. developed the idea and designed this study. W.H. and L.L. collected and verified the data. Y.R., Y.W., and H.C. did the analysis and prepared the manuscript. All authors critically revised the manuscript for important intellectual content and agreed to submit the final version for publication. All authors agree to be accountable for the work to ensure that questions related to the accuracy or integrity of any part of the work are appropriately investigated and resolved. All authors had full access to all the data in the study and had final responsibility for the decision to submit it for publication. The corresponding authors attest that all listed authors meet authorship criteria.

## Funding

This study was supported by the Noncommunicable Chronic Diseases–National Science and Technology Major Project (2023ZD0506200 and 2023ZD0506205).

## Ethics Statement

Approval of the study from the National Center of Health and Statistics Research Ethics Review Board was waived because the research relied on publicly used, de‐identified secondary data.

## Consent

This study utilized de‐identified, publicly available data from the National Health and Nutrition Examination Survey (NHANES), administered by the National Center for Health Statistics (NCHS), Centers for Disease Control and Prevention (CDC). As the data are fully anonymized and publicly accessible for research purposes, and no new human participants were recruited or interacted with for this specific analysis, obtaining additional individual consent for publication is not applicable. All analyses were conducted in accordance with the NHANES data use guidelines.

## Conflicts of Interest

The authors declare no conflicts of interest.

## Supporting information


**Table S1:** Laboratory and clinical characteristics of participants by spirometric pattern.

**Table S2:** Characteristics of PRISm participants by frailty status.

**Table S3:** Survey‐weighted logistic regression examining the association between frailty and PRISm.

**Table S4:** Survey‐weighted multivariable logistic regression analysis of factors associated with major adverse cardiovascular events (MACEs) in individuals with PRISm.

**Table S5:** Baseline characteristics of PRISm participants stratified by frailty severity (FI‐LAB, Set 2).

**Table S6:** Baseline characteristics of individuals with PRISm stratified by frailty severity.

**Table S7:** Survey‐weighted logistic regression examining the association between frailty and LLN‐defined PRISm.

**Table S8:** Multivariable logistic regression analysis for MACE in PRISm defined by LLN criteria.

**Table S9:** Multivariable Cox proportional hazards analysis for all‐cause mortality in PRISm defined by LLN criteria.

**Figure S1:** Supporting information.

**Figure S2:** Supporting information.

**Figure S3:** Supporting information.

**Figure S4:** Supporting information.

## Data Availability

The datasets generated and/or analyzed during the current study are publicly available from the National Health and Nutrition Examination Survey (NHANES), conducted by the National Center for Health Statistics (NCHS), Centers for Disease Control and Prevention (CDC). NHANES data are available at https://www.cdc.gov. The datasets supporting the findings of this study correspond to the NHANES cycles (2007–2012). Relevant data files, including demographics, questionnaire data, and laboratory data, were downloaded from the above website for analysis.
