# Supplementary material for: Frailty as a Key Determinant of Cardiovascular Risk and Mortality in Preserved Ratio Impaired Spirometry: A Nationally Representative Study
Source: Clin Respir J. 2026 Jan 10;20(1):e70165. doi: 10.1111/crj.70165 (PMC12790094; doi:10.1111/crj.70165)
Supplement: Supplementary file 1 — Table S1: Laboratory and clinical characteristics of participants by spirometric pattern. [file CRJ-20-e70165-s002.docx]

**Supplementary Table 1. Laboratory and Clinical Characteristics of Participants by Spirometric Pattern**

| **Characteristics** | **Total (N=8,882)** | **NS (N=8,119)** | **PRISm (N=763)** | **P value** |
| --- | --- | --- | --- | --- |
| **Weighted population** | 124,585,152 | 117,554,976 | 7,030,176 | — |
| **Blood pressure** |  |  |  |  |
| Systolic BP (mmHg) | 119.4 ± 15.4 | 119.1 ± 15.0 | 125.5 ± 19.0 | <0.0001 |
| Diastolic BP (mmHg) | 71.2 ± 11.8 | 71.2 ± 11.6 | 70.8 ± 14.7 | <0.0001 |
| **Lung function (predicted)** |  |  |  |  |
| Predicted FEV₁ (mL) | 3260.2 ± 744.4 | 3268.4 ± 741.3 | 3123.2 ± 780.9 | <0.0001 |
| **Hematologic tests** |  |  |  |  |
| WBC (×10³ cells/µL) | 7.1 ± 2.1 | 7.1 ± 2.1 | 7.2 ± 2.1 | <0.0001 |
| Hemoglobin (g/dL) | 14.3 ± 1.4 | 14.3 ± 1.4 | 13.9 ± 1.5 | <0.0001 |
| MCV (fL) | 89.2 ± 5.2 | 89.2 ± 5.1 | 88.2 ± 6.3 | <0.0001 |
| **Biochemistry** |  |  |  |  |
| Albumin (g/L) | 43.1 ± 3.2 | 43.2 ± 3.2 | 41.5 ± 3.1 | <0.0001 |
| ALP (U/L) | 65.5 ± 20.0 | 65.1 ± 20.0 | 71.4 ± 23.0 | <0.0001 |
| AST (U/L) | 25.9 ± 16.3 | 26.0 ± 16.6 | 25.3 ± 10.5 | <0.0001 |
| Total calcium (mmol/L) | 2.35 ± 0.09 | 2.35 ± 0.09 | 2.36 ± 0.09 | <0.0001 |
| Creatinine (µmol/L) | 76.0 ± 23.6 | 75.7 ± 21.4 | 81.6 ± 46.7 | <0.0001 |
| Bicarbonate (mmol/L) | 25.1 ± 2.1 | 25.0 ± 2.1 | 25.6 ± 2.3 | <0.0001 |
| Triglycerides (mmol/L) | 1.36 (0.89–2.12) | 1.36 (0.89–2.12) | 1.28 (0.87–2.05) | 0.072 |
| BUN (mmol/L) | 4.51 ± 1.61 | 4.51 ± 1.55 | 4.55 ± 2.34 | <0.0001 |
| GGT (U/L) | 19.0 (14–29) | 19.0 (14–29) | 22.0 (16–34) | <0.0001 |
| Glucose (mmol/L) | 5.4 ± 1.7 | 5.3 ± 1.6 | 6.2 ± 3.1 | <0.0001 |
| Iron (µmol/L) | 15.8 ± 6.5 | 15.9 ± 6.6 | 13.7 ± 5.3 | <0.0001 |
| LDH (U/L) | 128.0 ± 28.3 | 127.5 ± 28.2 | 135.9 ± 30.2 | <0.0001 |
| Phosphorus (mmol/L) | 1.21 ± 0.18 | 1.21 ± 0.18 | 1.20 ± 0.18 | <0.0001 |
| Total protein (g/L) | 71.3 ± 4.4 | 71.3 ± 4.4 | 71.7 ± 4.7 | <0.0001 |
| Sodium (mmol/L) | 139.1 ± 2.1 | 139.1 ± 2.1 | 139.3 ± 2.3 | <0.0001 |
| Potassium (mmol/L) | 3.9 ± 0.3 | 3.9 ± 0.3 | 4.0 ± 0.4 | <0.0001 |
| Serum folate (nmol/L) | 42.3 ± 24.3 | 42.5 ± 24.3 | 40.1 ± 24.6 | <0.0001 |
| RBC folate (nmol/L) | 1150.2 ± 477.8 | 1151.4 ± 473.8 | 1130.5 ± 540.0 | <0.001 |
| **Respiratory function** |  |  |  |  |
| Baseline PEF (mL/s) | 8628.9 ± 2092.9 | 8747.3 ± 2051.6 | 6649.2 ± 1757.1 | <0.0001 |
| Baseline FEF25–75% (mL/s) | 3284.6 ± 1158.9 | 3356.6 ± 1137.5 | 2080.8 ± 792.0 | <0.0001 |
| **Frailty groups** |  |  |  | <0.0001 |
| FI group 1 |  |  |  |  |
| Non-frail | 2.3% | 1.5% | — |  |
| Pre-frail | 64.7% | 55.3% | — |  |
| Frail | 33.0% | 43.1% | — |  |
| FI group 2 |  |  |  |  |
| Non-frail | 5.1% | 5.2% | 3.8% |  |
| Pre-frail | 49.0% | 49.4% | 42.3% |  |
| Frail | 44.7% | 44.3% | 51.5% |  |
| Severely frail | 1.2% | 1.1% | 2.4% |  |
| **Health status** |  |  |  | <0.0001 |
| Better than 1 year ago | 18.6% | 18.7% | 16.9% |  |
| Worse | 9.3% | 9.2% | 12.0% |  |
| Same | 72.0% | 72.1% | 71.1% |  |
| **Healthcare utilization** |  |  |  | <0.0001 |
| Overnight hospitalization | 7.6% | 7.3% | 11.9% |  |
| **Depression categories** |  |  |  | <0.0001 |
| No depression | 78.7% | 78.9% | 74.5% |  |
| Mild | 14.1% | 14.0% | 14.3% |  |
| Moderate | 4.5% | 4.4% | 6.5% |  |
| Moderately severe | 2.0% | 1.9% | 3.3% |  |
| Severe | 0.7% | 0.7% | 1.4% |  |
| **Comorbidities** |  |  |  | <0.0001 |
| Hypertension | 25.2% | 24.2% | 42.1% |  |
| Diabetes | 6.7% | 5.8% | 21.9% |  |
| Hypercholesterolemia | 36.3% | 36.2% | 38.8% |  |
| Heart failure | 1.0% | 0.8% | 4.5% |  |
| Coronary heart disease | 1.6% | 1.4% | 4.5% |  |
| Angina | 1.2% | 1.1% | 3.3% |  |
| MI | 1.5% | 1.4% | 4.4% |  |
| Stroke | 1.3% | 1.2% | 2.8% |  |
| **Lifestyle factors** |  |  |  | <0.0001 |
| Vigorous work activity | 21.4% | 21.7% | 16.2% |  |
| Moderate work activity | 41.0% | 41.2% | 37.6% |  |
| Vigorous recreational activity | 29.7% | 30.4% | 19.4% |  |
| Moderate recreational activity | 48.1% | 48.8% | 36.6% |  |

Abbreviations:

NS, normal spirometry; PRISm, Preserved Ratio Impaired Spirometry;

FEV₁, forced expiratory volume in 1 s; FVC, forced vital capacity;

BP, blood pressure; WBC, white blood cell count; MI: myocardial infarction

PEF, peak expiratory flow; FEF25–75%, forced expiratory flow 25–75%;

LDH, lactate dehydrogenase; BUN, blood urea nitrogen; GGT, γ-glutamyltransferase.

Values are weighted means ± standard deviations or weighted percentages unless otherwise indicated.Median (IQR) shown for skewed variables.
