# Supplementary material for: Frailty as a Key Determinant of Cardiovascular Risk and Mortality in Preserved Ratio Impaired Spirometry: A Nationally Representative Study
Source: Clin Respir J. 2026 Jan 10;20(1):e70165. doi: 10.1111/crj.70165 (PMC12790094; doi:10.1111/crj.70165)
Supplement: Supplementary file 2 — Table S2: Characteristics of PRISm participants by frailty status. [file CRJ-20-e70165-s005.docx]

**Supplementary Table 2. Characteristics of PRISm Participants by Frailty Status**

| **Characteristics** | **PRISm (N = 763)** | **Non-frail (N = 318)** | **Frail (N = 445)** | **P value** |
| --- | --- | --- | --- | --- |
| **Weighted population** | 7,030,176 | 3,238,451 | 3,791,725 | 0.5393 |
| **Age (years)** | 47.8 ± 14.5 | 45.9 ± 14.3 | 49.4 ± 15.3 | <0.0001 |
| **Gender, %** |  |  |  | <0.0001 |
| Male | 43.7 | 52.5 | 36.2 |  |
| Female | 56.3 | 47.5 | 63.8 |  |
| **BMI (kg/m²)** | 31.9 ± 8.7 | 30.9 ± 7.7 | 32.6 ± 9.4 | <0.0001 |
| **Race, %** |  |  |  | <0.0001 |
| Mexican American | 2.7 | 2.6 | 2.7 |  |
| Other Hispanic | 3.0 | 3.1 | 2.9 |  |
| Non-Hispanic White | 38.7 | 42.2 | 35.8 |  |
| Non-Hispanic Black | 45.1 | 40.3 | 49.2 |  |
| Other Race | 10.5 | 11.9 | 9.3 |  |
| **Education, %** |  |  |  | <0.0001 |
| Less than high school | 19.8 | 20.6 | 19.1 |  |
| High school or equivalent | 26.3 | 23.8 | 28.3 |  |
| Greater than high school | 53.9 | 55.5 | 52.5 |  |
| **Marital status, %** |  |  |  | <0.0001 |
| Married | 48.3 | 51.1 | 45.9 |  |
| Widowed / divorced / separated | 24.6 | 22.6 | 26.3 |  |
| Never married | 20.6 | 20.9 | 20.4 |  |
| Living with partner | 6.5 | 5.3 | 7.4 |  |
| **PIR** | 2.6 ± 1.7 | 2.8 ± 1.7 | 2.5 ± 1.6 | <0.0001 |
| **Baseline lung volumes** |  |  |  |  |
| Baseline FVC (mL) | 2952.8 ± 839.3 | 3134.8 ± 846.2 | 2797.4 ± 583.8 | <0.0001 |
| Baseline FEV₁ (mL) | 2292.3 ± 623.3 | 2434.0 ± 638.0 | 2171.3 ± 583.8 | <0.0001 |
| **Frailty index** | 0.24 ± 0.09 | 0.17 ± 0.04 | 0.31 ± 0.07 | <0.0001 |
| **Alcohol, %** |  |  |  | <0.0001 |
| Never drinkers | 15.2 | 14.5 | 15.2 |  |
| Non-drinkers (no alcohol last year) | 20.0 | 18.6 | 21.5 |  |
| Moderate drinkers | 59.9 | 62.6 | 57.8 |  |
| Heavy drinkers | 4.9 | 4.3 | 5.4 |  |
| **Smoking status, %** |  |  |  | <0.0001 |
| Never smokers | 51.7 | 57.6 | 46.6 |  |
| Former smokers | 24.5 | 21.6 | 27.0 |  |
| Current smokers | 21.6 | 20.8* / 27.0† | 24.5 |  |
| **Comorbidities, %** |  |  |  | <0.0001 |
| Hypertension | 42.1 | 33.5 | 49.3 |  |
| Hypercholesterolemia | 38.8 | 39.4 | 41.0 |  |
| Diabetes | 21.9 | 17.3 | 25.8 |  |
| Prediabetes | 7.1 | 5.6 | 8.6 |  |
| Weak / failing kidneys | 3.5 | 2.1 | 4.7 |  |
| Asthma | 17.6 | 17.2 | 17.9 |  |
| Anemia | 4.3 | 3.2 | 5.3 |  |
| Overweight (binary) | 44.5 | 36.4 | 51.4 |  |
| Arthritis | 26.5 | 21.9 | 30.3 |  |
| Gout | 2.9 | 2.8 | 5.7 |  |
| Congestive heart failure | 4.5 | 2.4 | 6.4 |  |
| Coronary heart disease | 4.5 | 2.9 | 5.8 |  |
| Angina | 3.3 | 1.0 | 5.2 |  |
| Myocardial infarction (MI) | 4.4 | 2.3 | 6.1 |  |
| Stroke | 2.8 | 1.3 | 4.1 |  |
| Emphysema | 1.1 | 1.7 | 0.6 |  |
| Thyroid problem | 11.8 | 11.0 | 12.5 |  |
| Chronic bronchitis | 5.2 | 2.7 | 7.3 |  |
| Liver problem | 2.7 | 1.1 | 4.2 |  |
| Cancer / malignancy | 6.1 | 5.9 | 6.2 |  |
| **Mortality (%)** | 11.4 | 7.0 | 15.2 | <0.0001 |
| **Blood pressure in PRISm** |  |  |  |  |
| Systolic BP (mmHg) | 125.5 ± 19.0 | 121.8 ± 15.1 | 128.6 ± 21.4 | <0.0001 |
| Diastolic BP (mmHg) | 70.8 ± 14.7 | 70.8 ± 12.8 | 70.7 ± 16.1 | <0.0001 |
| **Predicted FEV₁ (mL)** | 3123.2 ± 780.9 | 3279.9 ± 816.3 | 2989.3 ± 722.8 | <0.0001 |
| **Selected labs** |  |  |  |  |
| WBC (×10³ cells/µL) | 7.2 ± 2.1 | 6.7 ± 1.7 | 7.7 ± 2.3 | <0.0001 |
| Hemoglobin (g/dL) | 13.9 ± 1.5 | 14.3 ± 1.3 | 13.5 ± 1.6 | <0.0001 |
| MCV (fL) | 88.2 ± 6.3 | 88.9 ± 5.1 | 87.6 ± 7.2 | <0.0001 |
| Albumin (g/L) | 41.5 ± 3.1 | 42.0 ± 2.7 | 41.1 ± 3.4 | <0.0001 |
| ALP (U/L) | 71.4 ± 23.0 | 69.3 ± 20.6 | 73.3 ± 24.7 | <0.0001 |
| AST (U/L) | 25.3 ± 10.5 | 24.7 ± 8.4 | 25.8 ± 12.0 | <0.0001 |
| Total calcium (mmol/L) | 2.36 ± 0.09 | 2.37 ± 0.08 | 2.34 ± 0.10 | <0.0001 |
| Creatinine (µmol/L) | 81.6 ± 46.7 | 77.5 ± 16.4 | 85.0 ± 61.6 | <0.0001 |
| Bicarbonate (mmol/L) | 25.6 ± 2.3 | 25.9 ± 2.3 | 25.3 ± 2.3 | <0.0001 |
| BUN (mmol/L) | 4.55 ± 2.34 | 4.42 ± 1.24 | 4.66 ± 2.97 | <0.0001 |
| GGT (U/L) | 22.0 (16.0–34.0) | 23.0 (16.0–33.0) | 23.0 (17.0–38.0) | 0.219 |
| Glucose (mmol/L) | 6.2 ± 3.1 | 5.7 ± 2.1 | 6.5 ± 3.7 | <0.0001 |
| Iron (µmol/L) | 13.7 ± 5.3 | 14.9 ± 5.3 | 12.7 ± 5.2 | <0.0001 |
| LDH (U/L) | 135.9 ± 30.2 | 134.0 ± 30.5 | 137.4 ± 29.9 | <0.0001 |
| Phosphorus (mmol/L) | 1.20 ± 0.18 | 1.20 ± 0.16 | 1.21 ± 0.19 | <0.0001 |
| Total protein (g/L) | 71.7 ± 4.7 | 71.5 ± 4.1 | 72.0 ± 5.2 | <0.0001 |
| Sodium (mmol/L) | 139.3 ± 2.3 | 139.6 ± 1.7 | 139.1 ± 2.7 | <0.0001 |
| Potassium (mmol/L) | 4.0 ± 0.4 | 3.99 ± 0.28 | 3.98 ± 0.44 | <0.0001 |
| Serum folate (nmol/L) | 40.1 ± 24.6 | 35.9 ± 18.4 | 43.6 ± 28.4 | <0.0001 |
| RBC folate (nmol/L) | 1130.5 ± 540.0 | 1019.1 ± 418.9 | 1224.7 ± 608.6 | <0.001 |
| Depression score (median, IQR) | 2 (0–5) | 2 (0–4.5) | 2 (0–5) | 0.059 |
| **Respiratory flow** |  |  |  |  |
| Baseline PEF (mL/s) | 6649.2 ± 1757.1 | 6968.7 ± 1710.8 | 6376.4 ± 1750.0 | <0.0001 |
| Baseline FEF25–75% (mL/s) | 2080.8 ± 792.0 | 2216.3 ± 807.7 | 1965.0 ± 759.5 | <0.0001 |
| **Other variables** |  |  |  |  |
| Globulin (g/L) | 30.2 ± 4.9 | 29.5 ± 4.2 | 30.9 ± 5.3 | <0.0001 |
| Bilirubin, total (µmol/L) | 11.7 ± 4.3 | 12.2 ± 4.3 | 11.3 ± 4.3 | <0.0001 |
| Uric acid (µmol/L) | 330.8 ± 86.9 | 333.3 ± 84.4 | 328.7 ± 88.9 | <0.0001 |
| Cotinine (ng/mL) | 0.102 (0.025–88.95) | 0.099 (0.024–98.0) | 0.106 (0.027–78.78) | 0.984 |
| **Health status compared with 1 year ago, %** |  |  |  | <0.0001 |
| Better | 16.9 | 15.3 | 18.4 |  |
| Worse | 12.0 | 10.5 | 13.2 |  |
| Same | 71.1 | 74.2 | 68.5 |  |
| **Overnight hospital last year** | 11.9 | 8.9 | 14.5 | <0.0001 |
| **Depression categories, %** |  |  |  | <0.0001 |
| No depression | 74.5 | 76.3 | 72.0 |  |
| Mild depression | 14.3 | 11.8 | 16.3 |  |
| Moderate depression | 6.5 | 7.8 | 5.9 |  |
| Moderately severe depression | 3.3 | 2.6 | 4.4 |  |
| Severe depression | 1.4 | 1.5 | 1.5 |  |
| **Activity & exposures, %** |  |  |  |  |
| Vigorous work activity | 16.2 | 16.6 | 15.8 | <0.0001 |
| Moderate work activity | 37.6 | 39.0 | 36.3 | <0.0001 |
| Vigorous recreational activity | 19.4 | 24.2 | 15.3 | <0.0001 |
| Moderate recreational activity | 36.6 | 38.2 | 35.2 | <0.0001 |
| Work exposure to mineral dusts | 26.4 | 27.9 | 24.9 | <0.0001 |
| Work exposure to organic dusts | 19.9 | 19.6 | 20.5 | <0.0001 |
| Work exposed to exhaust fumes | 22.0 | 21.9 | 21.9 | 0.340 |
| Other fumes | 27.5 | 29.7 | 25.6 | <0.0001 |
| **Age group, %** |  |  |  | <0.0001 |
| 20–39 years | 30.9 | 33.0 | 29.1 |  |
| 40–59 years | 44.6 | 46.6 | 42.9 |  |
| 60–79 years | 24.6 | 20.4 | 28.1 |  |
| **PIR group, %** |  |  |  | <0.0001 |
| Low income | 19.1 | 18.8 | 20.7 |  |
| Middle income | 53.2 | 45.1 | 55.4 |  |
| High income | 27.7 | 36.1 | 23.9 |  |
| **BMI group, %** |  |  |  | <0.0001 |
| Normal | 19.6 | 29.7 | 19.6 |  |
| Underweight | 2.3 | 1.9 | 2.6 |  |
| Overweight | 22.7 | 28.8 | 17.5 |  |
| Obesity | 55.4 | 48.7 | 61.1 |  |
| **FENO group, %** |  |  |  | <0.0001 |
| Low | 82.6 | 82.2 | 83.4 |  |
| Medium | 13.5 | 14.8 | 12.1 |  |
| High | 4.0 | 3.0 | 4.5 |  |
| **Ever received blood transfusion, %** | 13.7 | 8.5 | 18.1 | <0.0001 |
| **Healthcare visits in past year, %** |  |  |  | <0.0001 |
| None | 13.9 | 16.5 | 11.6 |  |
| 1 visit | 16.4 | 20.3 | 13.1 |  |
| 2–3 visits | 28.7 | 30.6 | 27.0 |  |
| 4–9 visits | 29.1 | 23.6 | 33.8 |  |
| 10–12 visits | 5.8 | 4.4 | 7.0 |  |
| ≥13 visits | 6.1 | 4.6 | 7.4 |  |
| **MACE (%)** | 16.2 | 9.7 | 21.6 | <0.0001 |

Abbreviations: PRISm = Preserved Ratio Impaired Spirometry; FEV₁ = forced expiratory volume in 1 s; FVC = forced vital capacity; PIR = poverty–income ratio; PEF = peak expiratory flow; FEF25–75% = forced expiratory flow at 25–75%; WBC = white blood cell count; BUN = blood urea nitrogen; GGT = γ-glutamyltransferase; LDH = lactate dehydrogenase; MACE =
