# Supplementary material for: Frailty as a Key Determinant of Cardiovascular Risk and Mortality in Preserved Ratio Impaired Spirometry: A Nationally Representative Study
Source: Clin Respir J. 2026 Jan 10;20(1):e70165. doi: 10.1111/crj.70165 (PMC12790094; doi:10.1111/crj.70165)
Supplement: Supplementary file 4 — Table S4: Survey‐weighted multivariable logistic regression analysis of factors associated with major adverse cardiovascular events (MACEs) in individuals with PRISm. [file CRJ-20-e70165-s007.docx]

Supplementary Table 4. Survey-weighted multivariable logistic regression analysis of factors associated with major adverse cardiovascular events (MACEs) in individuals with PRISm

| **Variable** | **B** | **P value** | **OR** | **95% CI (Lower)** | **95% CI (Upper)** |
| --- | --- | --- | --- | --- | --- |
| **Age (years)** | 0.068 | <0.001 | 1.071 | 1.051 | 1.091 |
| **Gamma-glutamyl transferase (U/L)** | 0.003 | 0.238 | 1.003 | 0.998 | 1.008 |
| **LDH (U/L)** | 0.009 | 0.008 | 1.009 | 1.002 | 1.016 |
| **Frailty index** | 2.938 | 0.018 | 18.87 | 1.653 | 215.476 |
| **Sex (female vs male)** | 0.583 | 0.017 | 1.791 | 1.110 | 2.889 |
| **Education** |  | 0.048 |  |  |  |
| High school vs <high school | 0.249 | 0.384 | 1.283 | 0.732 | 2.248 |
| >High school vs <high school | –0.553 | 0.079 | 0.575 | 0.310 | 1.065 |
| Poverty–income ratio | –0.115 | 0.174 | 0.891 | 0.754 | 1.052 |
| Mean cell hemoglobin (pg) | 0.077 | 0.057 | 1.080 | 0.998 | 1.169 |
| Bicarbonate (mmol/L) | –0.062 | 0.212 | 0.940 | 0.852 | 1.036 |
| **General health condition** |  | 0.003 |  |  |  |
| Very good/good vs excellent | –1.519 | 0.014 | 0.219 | 0.066 | 0.731 |
| Fair vs excellent | –1.303 | 0.001 | 0.272 | 0.127 | 0.581 |
| Poor vs excellent | –0.714 | 0.071 | 0.489 | 0.226 | 1.062 |
| **Anemia** | 1.100 | 0.023 | 3.004 | 1.166 | 7.742 |
| **Gout** | 0.326 | 0.429 | 1.385 | 0.617 | 3.106 |
| **Emphysema** | 2.190 | 0.014 | 8.937 | 1.571 | 50.861 |
| **Lymphocyte percent (%)** | –0.013 | 0.337 | 0.988 | 0.963 | 1.013 |
| **Monocyte number (10³ cells/µL)** | –0.127 | 0.848 | 0.881 | 0.240 | 3.234 |
| **Cholesterol (mmol/L)** | –0.109 | 0.332 | 0.897 | 0.720 | 1.117 |

**Abbreviations:** OR = odds ratio; CI = confidence interval; LDH = lactate dehydrogenase; MACE = major adverse cardiovascular event; PRISm = preserved ratio impaired spirometry; PIR = poverty–income ratio.
