# Supplementary material for: Frailty as a Key Determinant of Cardiovascular Risk and Mortality in Preserved Ratio Impaired Spirometry: A Nationally Representative Study
Source: Clin Respir J. 2026 Jan 10;20(1):e70165. doi: 10.1111/crj.70165 (PMC12790094; doi:10.1111/crj.70165)
Supplement: Supplementary file 5 — Table S5: Baseline characteristics of PRISm participants stratified by frailty severity (FI‐LAB, Set 2). [file CRJ-20-e70165-s013.docx]

Supplementary Table 5 Baseline characteristics of PRISm participants stratified by frailty severity (FI-LAB, Set 2)

| Characteristics | PRISm (N=763) | Non-frail (N=10) | Pre-frail (N=389) | Frail (N=364) | P value |
| --- | --- | --- | --- | --- | --- |
| Weighted population | 7,030,176 | 41,957 | 1,312,872 | 816,557 | — |
| **Age, years** | 47.8 ± 14.5 | 40.75 ± 11.82 | 46.38 ± 14.6 | 49.4 ± 15.1 | <0.0001 |
| **Sex** |  |  |  |  | <0.0001 |
| **Male** | 43.70% | 82.70% | 51.50% | 32.30% |  |
| **Female** | 56.30% | 17.30% | 48.50% | 67.70% |  |
| **BMI, kg/m²** | 31.9 ± 8.7 | 34.6 ± 6.7 | 30.7 ± 7.5 | 33.3 ± 9.9 | <0.0001 |
| **Race / ethnicity** |  |  |  |  | <0.0001 |
| Mexican American | 2.70% | 3.10% | 2.50% | 2.80% |  |
| Other Hispanic | 3.00% | 0.00% | 4.10% | 1.80% |  |
| Non-Hispanic White | 38.70% | 29.20% | 42.20% | 34.60% |  |
| Non-Hispanic Black | 45.10% | 67.60% | 39.50% | 51.50% |  |
| Other race | 10.50% | 0.00% | 11.70% | 9.30% |  |
| **Education** |  |  |  |  | <0.0001 |
| Less than high school | 19.80% | 22.30% | 20.10% | 19.30% |  |
| High school or equivalent | 26.30% | 17.50% | 25.70% | 27.30% |  |
| Greater than high school | 53.90% | 60.10% | 54.20% | 53.40% |  |
| **Marital status** |  |  |  |  | <0.0001 |
| Married | 48.30% | 44.20% | 50.10% | 46.10% |  |
| Widowed/divorced/separated | 24.60% | 17.30% | 23.30% | 26.60% |  |
| Never married | 20.60% | 17.50% | 21.50% | 19.60% |  |
| Living with partner | 6.50% | 21.00% | 5.10% | 7.70% |  |
| **FVC, mL** | 2952.8 ± 839.3 | 3675.1 ± 675.2 | 3093.5 ± 842.6 | 2746.6 ± 583.8 | <0.0001 |
| **FEV1, mL** | 2292.3 ± 623.3 | 2783.8 ± 458.1 | 2401.4 ± 635.3 | 2134.9 ± 571.6 | <0.0001 |
| **Frailty index** | 0.24 ± 0.09 | 0.05 ± 0.00 | 0.18 ± 0.05 | 0.33 ± 0.07 | <0.0001 |
| **Alcohol consumption** |  |  |  |  | <0.0001 |
| Never drinkers | 15.20% | 9.10% | 14.10% | 16.10% |  |
| Non-drinkers | 20.00% | 29.20% | 18.30% | 22.20% |  |
| Moderate drinkers | 59.90% | 61.70% | 64.00% | 54.80% |  |
| Heavy drinkers | 4.90% | 0.00% | 3.60% | 6.80% |  |
| **Smoking status** |  |  |  |  | <0.0001 |
| Never smokers | 51.70% | 56.50% | 53.20% | 49.60% |  |
| Former smokers | 23.80% | 33.30% | 21.90% | 25.90% |  |
| Current smokers | 24.50% | 10.10% | 24.90% | 24.50% |  |
| **Hypertension** | 42.10% | 28.10% | 34.90% | 51.70% | <0.0001 |
| **Hypercholesterolemia** | 38.80% | 39.40% | — | 41.00% | <0.0001 |
| **Diabetes** | 21.90% | 11.80% | 18.90% | 26.00% | <0.0001 |
| **Prediabetes** | 7.10% | 0.00% | 6.70% | 8.00% | <0.0001 |
| **General health status** |  |  |  |  | <0.0001 |
| Excellent | 11.10% | 10.00% | 13.70% | 7.70% |  |
| Very good / good | 62.40% | 62.50% | 63.90% | 60.50% |  |
| Fair | 21.10% | 27.50% | 17.90% | 25.00% |  |
| Poor | 5.40% | 0.00% | 4.50% | 6.80% |  |
| **Chronic kidney disease** | 3.50% | 0.00% | 2.10% | 5.40% | <0.0001 |
| **Asthma** | 17.60% | 44.90% | 16.50% | 18.00% | <0.0001 |
| **Anemia** | 4.30% | 0.00% | 3.00% | 6.20% | <0.0001 |
| **Arthritis** | 26.50% | 4.10% | 23.70% | 30.80% | <0.0001 |
| **Gout** | 2.90% | 0.00% | 3.00% | 6.40% | <0.0001 |
| **Congestive heart failure** | 4.50% | 0.00% | 3.20% | 6.50% | <0.0001 |
| **Coronary heart disease** | 4.50% | 0.00% | 4.50% | 4.60% | <0.0001 |
| **Angina** | 3.30% | 0.00% | 1.90% | 5.30% | <0.0001 |
| **Myocardial infarction** | 4.40% | 0.00% | 3.20% | 5.90% | <0.0001 |
| **Stroke** | 2.80% | 0.00% | 1.00% | 5.10% | <0.0001 |
| **Emphysema** | 1.10% | 0.00% | 1.80% | 0.20% | <0.0001 |
| **Thyroid disease** | 11.80% | 0.00% | 10.30% | 14.10% | <0.0001 |
| **Chronic bronchitis** | 5.20% | 0.00% | 3.10% | 8.10% | <0.0001 |
| **Cancer / malignancy** | 6.10% | 0.00% | 6.00% | 6.40% | <0.0001 |
| **All-cause mortality** | 15.20% | 9.10% | 8.10% | 15.80% | <0.0001 |

Data are presented as weighted mean ± standard deviation for continuous variables and weighted percentages for categorical variables. All analyses accounted for NHANES sampling weights, strata, and primary sampling units to ensure national representativeness.

Frailty was defined using a laboratory-based frailty index (FI-LAB, Set 2) and categorized as non-frail, pre-frail, and frail according to established cut-offs. PRISm was defined using lower limit of normal (LLN) criteria from pre-bronchodilator spirometry.

P values were obtained from survey-weighted linear regression for continuous variables and Rao–Scott chi-square tests for categorical variables; all tests were two-sided with P < 0.05 considered significant. “—” indicates unavailable or insufficient data.

Abbreviations: PRISm, preserved ratio impaired spirometry; BMI, body mass index; FVC, forced vital capacity; FEV₁, forced expiratory volume in 1 second; FI, frailty index.
