# Supplementary material for: Frailty as a Key Determinant of Cardiovascular Risk and Mortality in Preserved Ratio Impaired Spirometry: A Nationally Representative Study
Source: Clin Respir J. 2026 Jan 10;20(1):e70165. doi: 10.1111/crj.70165 (PMC12790094; doi:10.1111/crj.70165)
Supplement: Supplementary file 6 — Table S6: Baseline characteristics of individuals with PRISm stratified by frailty severity. [file CRJ-20-e70165-s004.docx]

Supplementary Table 6 Baseline characteristics of individuals with PRISm stratified by frailty severity

| Characteristics | PRISm (N=763) | Non-frail (N=25) | Pre-frail (N=293) | Frail (N=420) | Severely frail (N=25) | P value |
| --- | --- | --- | --- | --- | --- | --- |
| Weighted population | 7,030,176 | 264,638 | 2,973,813 | 3,619,775 | 171,949 | — |
| Age, years | 47.8 ± 14.5 | 44.3 ± 11.4 | 46.1 ± 14.5 | 49.4 ± 15.3 | 50.2 ± 15.7 | <0.0001 |
| Sex |  |  |  |  |  | <0.0001 |
| Male | 43.7% | 76.4% | 50.4% | 36.3% | 34.2% |  |
| Female | 56.3% | 23.6% | 49.6% | 63.7% | 65.8% |  |
| BMI, kg/m² | 31.9 ± 8.7 | 33.7 ± 6.8 | 32.6 ± 9.5 | 33.4 ± 6.8 | — | <0.0001 |
| Race / ethnicity |  |  |  |  |  | <0.0001 |
| Mexican American | 2.7% | 4.3% | 2.4% | 2.8% | 1.4% |  |
| Other Hispanic | 3.0% | 2.3% | 3.2% | 2.6% | 10.2% |  |
| Non-Hispanic White | 38.7% | 39.8% | 42.4% | 36.0% | 31.3% |  |
| Non-Hispanic Black | 45.1% | 53.7% | 39.1% | 49.2% | 50.7% |  |
| Other race | 10.5% | 0.0% | 12.9% | 9.5% | 6.3% |  |
| Education |  |  |  |  |  | <0.0001 |
| Less than high school | 19.8% | 29.3% | 19.9% | 19.0% | 21.3% |  |
| High school or equivalent | 26.3% | 7.2% | 25.3% | 28.6% | 23.6% |  |
| Greater than high school | 53.9% | 63.5% | 54.8% | 52.4% | 55.0% |  |
| Marital status |  |  |  |  |  | <0.0001 |
| Married | 48.3% | 51.0% | 51.1% | 45.7% | 49.4% |  |
| Widowed/divorced/separated | 24.6% | 25.9% | 22.3% | 25.7% | 39.5% |  |
| Never married | 20.6% | 13.7% | 21.6% | 20.9% | 8.9% |  |
| Living with partner | 6.5% | 9.3% | 5.0% | 7.7% | 2.2% |  |
| FVC, mL | 2952.8 ± 839.3 | 3615.6 ± 767.3 | 3092.1 ± 839.6 | 2799.9 ± 762.2 | 2744.1 ± 738.3 | <0.0001 |
| FEV₁, mL | 2292.3 ± 623.3 | 2733.3 ± 503.9 | 2407.4 ± 641.9 | 2171.2 ± 583.3 | 2174.3 ± 594.5 | <0.0001 |
| PEF, mL/s | 6649.2 ± 1757.1 | 7512.4 ± 1609.5 | 6920.3 ± 1711.2 | 6370.6 ± 1724.9 | 6497.9 ± 2210.4 | <0.0001 |
| FEF25–75%, mL/s | 2080.8 ± 792.0 | 2275.5 ± 450.4 | 2211.0 ± 831.9 | 1956.0 ± 754.0 | 2156.0 ± 842.9 | <0.0001 |
| Frailty index | 0.24 ± 0.09 | 0.08 ± 0.02 | 0.17 ± 0.03 | 0.30 ± 0.06 | 0.50 ± 0.05 | <0.0001 |
| Hypertension | 42.1% | 33.8% | 33.5% | 48.7% | 63.9% | <0.0001 |
| Diabetes | 21.9% | 24.8% | 16.6% | 25.1% | 40.5% | <0.0001 |
| Congestive heart failure | 4.5% | 0.0% | 2.6% | 6.2% | 9.4% | <0.0001 |
| Coronary heart disease | 4.5% | 0.0% | 3.1% | 5.2% | 18.5% | <0.0001 |
| Stroke | 2.8% | 2.3% | 1.2% | 4.3% | 0.0% | <0.0001 |
| All-cause mortality | 11.4% | 7.1% | 7.0% | 14.9% | 22.3% | <0.0001 |

Data are presented as mean ± standard deviation for continuous variables and percentage (%) for categorical variables, unless otherwise indicated.

Weighted population estimates were calculated according to the complex, multistage probability sampling design of the National Health and Nutrition Examination Survey (NHANES).

Frailty severity was defined using the laboratory-based frailty index (FI-LAB) and categorized as non-frail, pre-frail, frail, and severely frail according to established thresholds.

P values were obtained using survey-weighted linear regression for continuous variables and survey-weighted chi-square tests for categorical variables.

Abbreviations: PRISm, preserved ratio impaired spirometry; BMI, body mass index; FVC, forced vital capacity; FEV₁, forced expiratory volume in 1 second; PEF, peak expiratory flow; FEF25–75%, forced expiratory flow at 25–75% of FVC; PIR, poverty–income ratio; FI-LAB, laboratory-based frailty index.
