# Supplementary material for: Frailty as a Key Determinant of Cardiovascular Risk and Mortality in Preserved Ratio Impaired Spirometry: A Nationally Representative Study
Source: Clin Respir J. 2026 Jan 10;20(1):e70165. doi: 10.1111/crj.70165 (PMC12790094; doi:10.1111/crj.70165)
Supplement: Supplementary file 7 — Table S7: Survey‐weighted logistic regression examining the association between frailty and LLN‐defined PRISm. [file CRJ-20-e70165-s010.docx]

Supplementary Table 7. Survey-weighted logistic regression examining the association between frailty and LLN-defiend PRISm

| **Outcome** | **Exposure** | **Reference** | **OR** | **95% CI** | **P value** |
| --- | --- | --- | --- | --- | --- |
| **Outcome: PRISm** |  |  |  |  |  |
| Unadjusted | Frailty | Non-frail | 1.332 | 1.327–1.338 | <0.001 |
| Model 1 | Frailty | Non-frail | 1.217 | 1.212–1.223 | <0.001 |
| Model 2 | Frailty | Non-frail | 1.01 | 1.005–1.015 | <0.001 |
| **Outcome: Frailty** |  |  |  |  |  |
| Unadjusted | PRISm | NS | 1.069 | 1.068–1.070 | <0.001 |
| Model 1 | PRISm | NS | 1.076 | 1.075–1.077 | <0.001 |
| Model 2 | PRISm | NS | 1.052 | 1.051–1.053 | <0.001 |

Unadjusted model: univariable survey-weighted logistic regression.

Model 1: adjusted for age, sex, race, and BMI.

Model 2: adjusted for age, sex, race, BMI, education, marital status, PIR, smoking status,and alcohol intake.
