# Supplementary material for: Frailty as a Key Determinant of Cardiovascular Risk and Mortality in Preserved Ratio Impaired Spirometry: A Nationally Representative Study
Source: Clin Respir J. 2026 Jan 10;20(1):e70165. doi: 10.1111/crj.70165 (PMC12790094; doi:10.1111/crj.70165)
Supplement: Supplementary file 8 — Table S8: Multivariable logistic regression analysis for MACE in PRISm defined by LLN criteria. [file CRJ-20-e70165-s001.docx]

Supplementary Table 8. Multivariable Logistic Regression Analysis for MACE in PRISm Defined by LLN Criteria

| **Variable** | **OR** | **95% CI** | **P value** |
| --- | --- | --- | --- |
| Age (per year) | 1.082 | 1.082–1.082 | <0.001 |
| Frailty index | 50.758 | 50.225–51.297 | <0.001 |
| Male sex | 2.549 | 2.544–2.554 | <0.001 |
| Anemia (yes) | 2.185 | 2.175–2.196 | <0.001 |
| Emphysema (yes) | 2.855 | 2.836–2.874 | <0.001 |

Values are presented as odds ratios (ORs) with 95% confidence intervals (CIs). Multivariable logistic regression was performed using survey-weighted analyses. PRISm was defined according to lower limit of normal (LLN) criteria. Abbreviations: PRISm, preserved ratio impaired spirometry; LLN, lower limit of normal; OR, odds ratio; CI, confidence interval; MACE, major adverse cardiovascular events.
