# Supplementary material for: Frailty as a Key Determinant of Cardiovascular Risk and Mortality in Preserved Ratio Impaired Spirometry: A Nationally Representative Study
Source: Clin Respir J. 2026 Jan 10;20(1):e70165. doi: 10.1111/crj.70165 (PMC12790094; doi:10.1111/crj.70165)
Supplement: Supplementary file 9 — Table S9: Multivariable Cox proportional hazards analysis for all‐cause mortality in PRISm defined by LLN criteria. [file CRJ-20-e70165-s003.docx]

Supplementary Table 9. Multivariable Cox Proportional Hazards Analysis for All-Cause Mortality in PRISm Defined by LLN Criteria

| **Variable** | **HR** | **95% CI** | **P value** |
| --- | --- | --- | --- |
| Frailty index | 26.92 | 8.38–86.53 | <0.001 |
| Age (per year) | 1.07 | 1.06–1.08 | <0.001 |
| Myocardial infarction | 1.28 | 0.81–2.01 | 0.291 |
| Heart failure | 2.90 | 1.89–4.46 | <0.001 |

Values are presented as hazard ratios (HRs) with 95% confidence intervals (CIs). Survey-weighted multivariable Cox proportional hazards regression was performed to examine associations with all-cause mortality among individuals with PRISm defined by lower limit of normal (LLN) spirometric criteria. A two-sided P value < 0.05 was considered statistically significant.
