# Supplementary figures and images for: Frailty as a Key Determinant of Cardiovascular Risk and Mortality in Preserved Ratio Impaired Spirometry: A Nationally Representative Study
Source: Clin Respir J. 2026 Jan 10;20(1):e70165. doi: 10.1111/crj.70165 (PMC12790094; doi:10.1111/crj.70165)

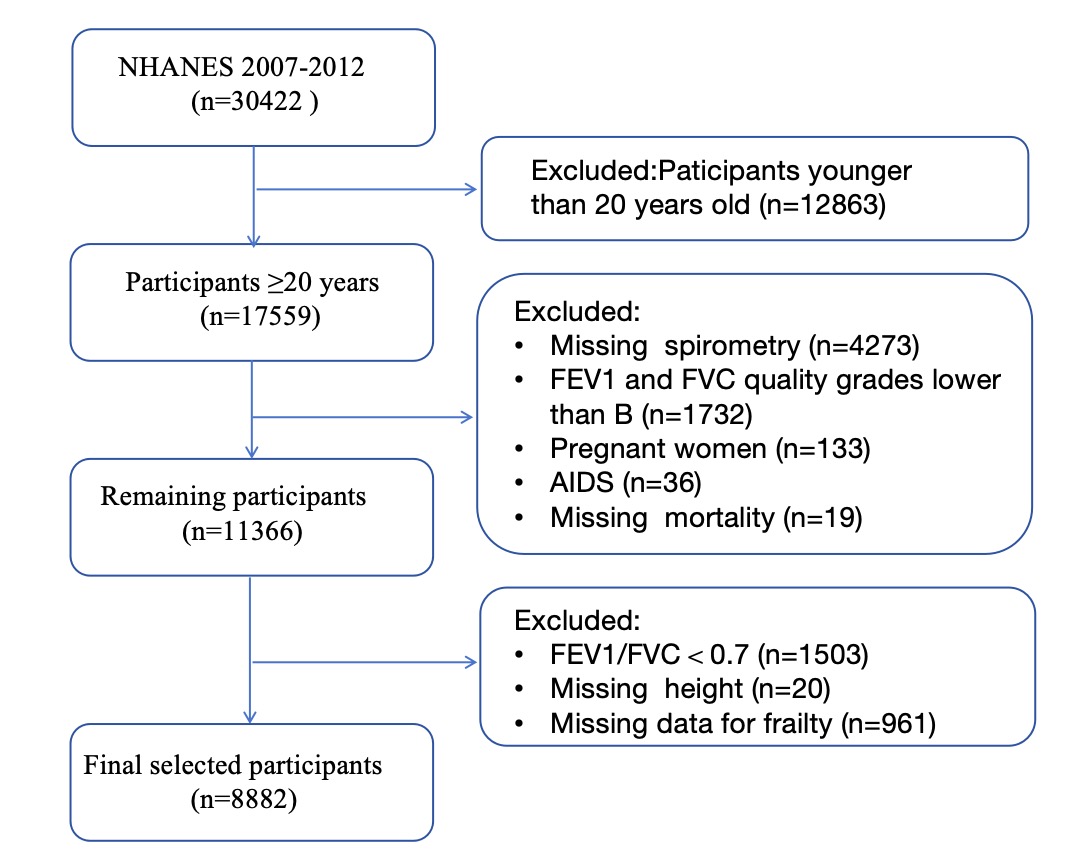

Supplement: Supplementary file 10 — Figure S1: Supporting information. [file CRJ-20-e70165-s008.jpg]

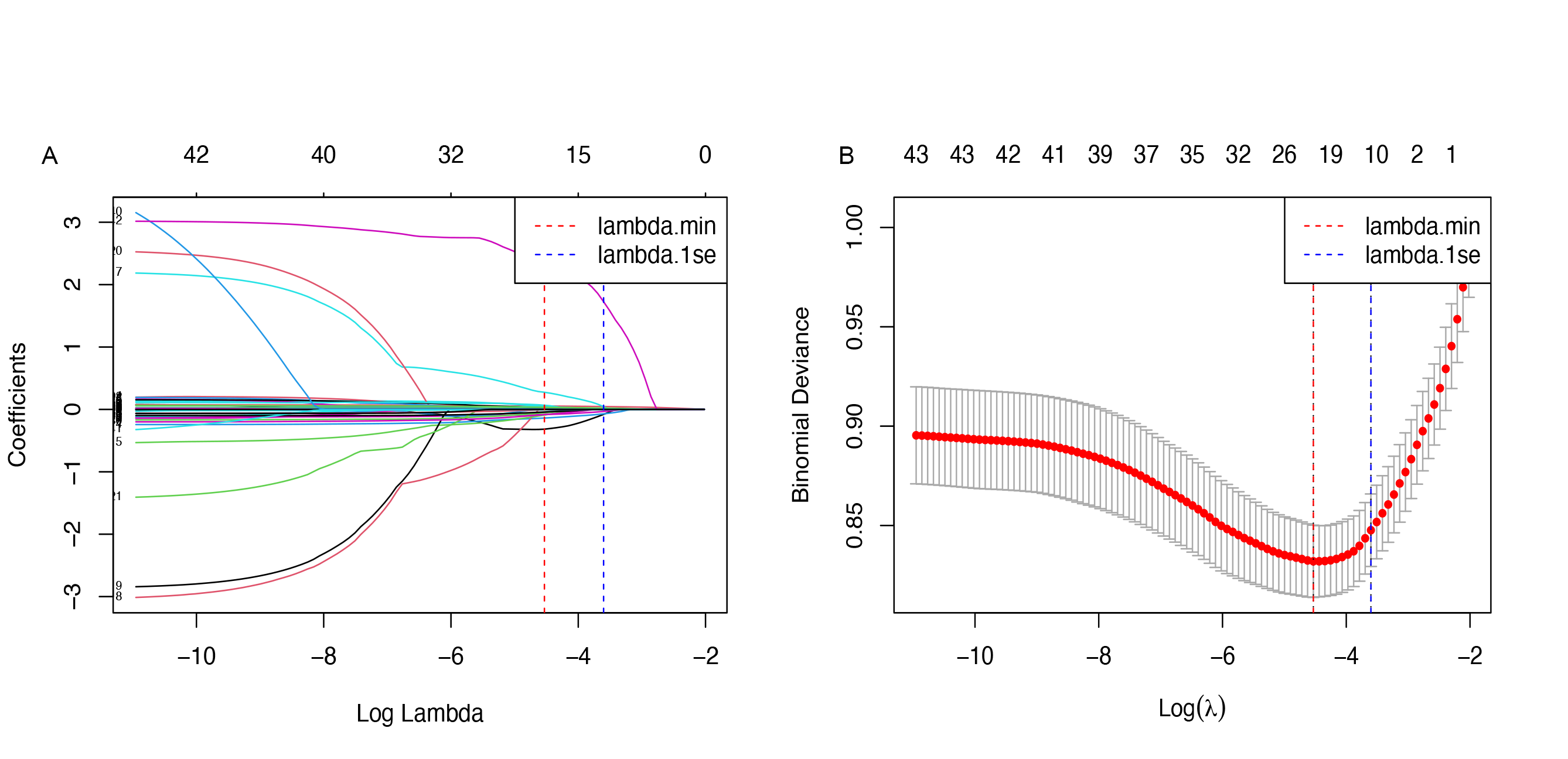

Supplement: Supplementary file 11 — Figure S2: Supporting information. [file CRJ-20-e70165-s006.png]

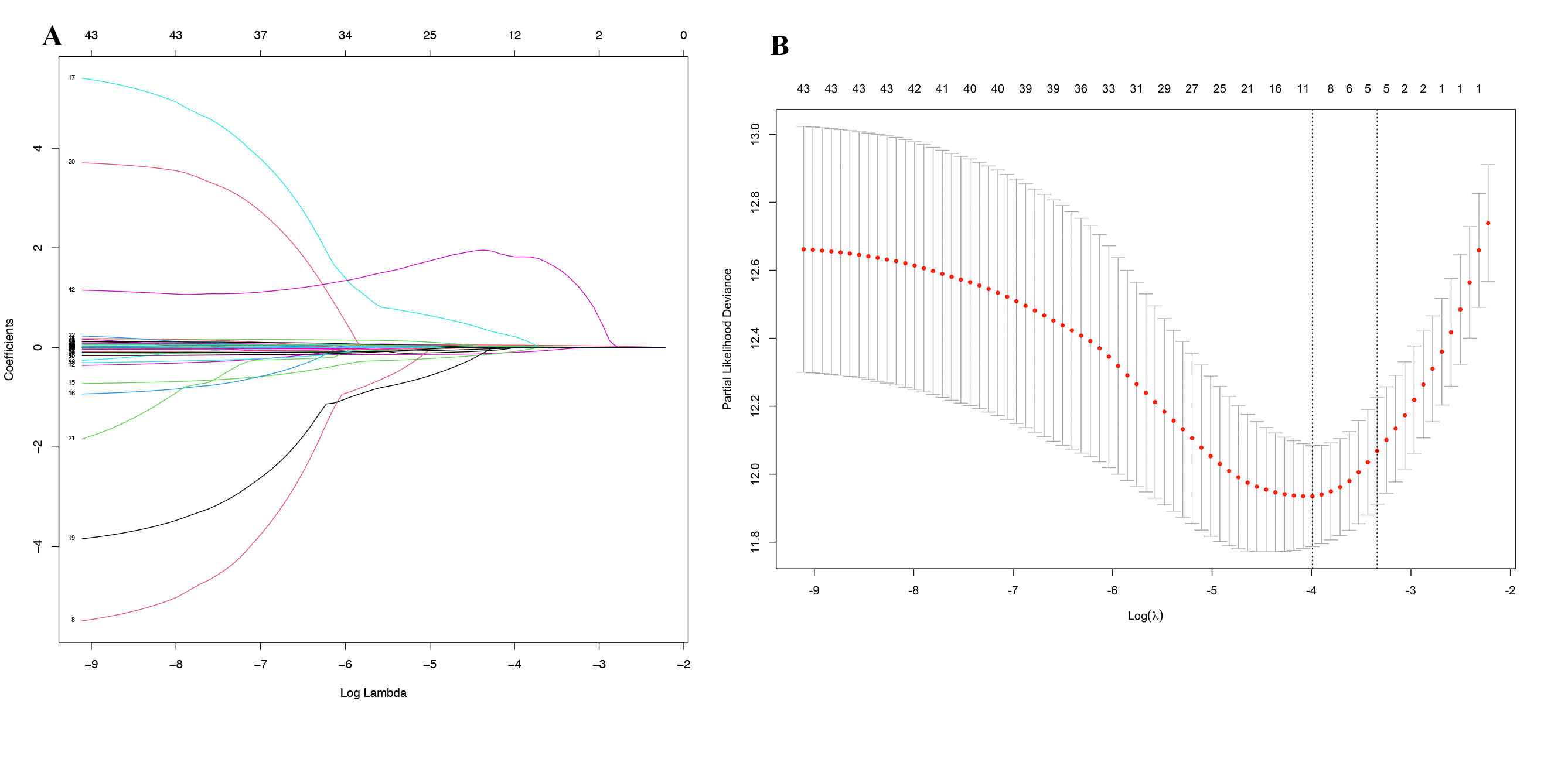

Supplement: Supplementary file 12 — Figure S3: Supporting information. [file CRJ-20-e70165-s012.png]

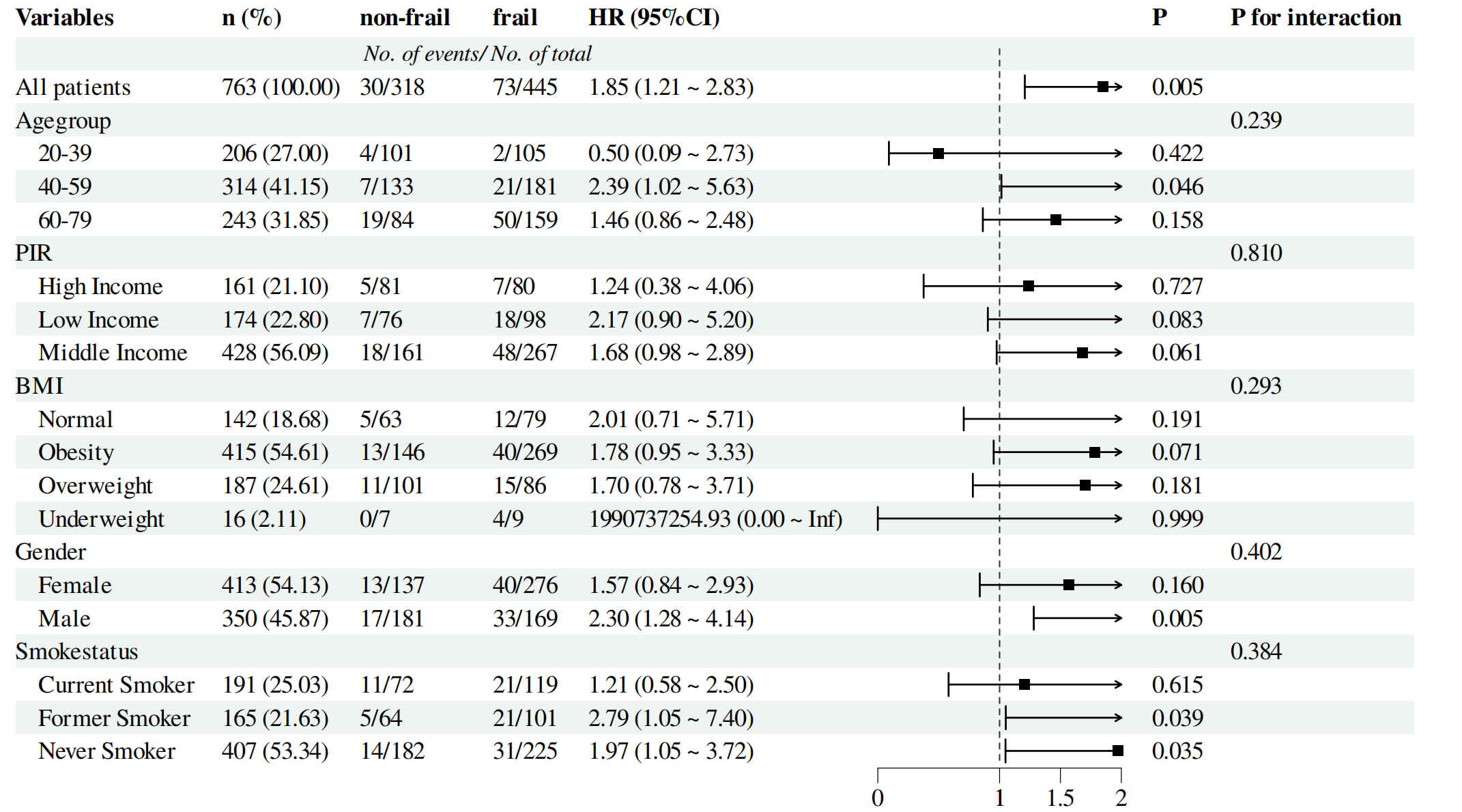

Supplement: Supplementary file 13 — Figure S4: Supporting information. [file CRJ-20-e70165-s009.png]
